# mTORC1-Dependent Protein and Parkinson’s Disease: A Mendelian Randomization Study

**DOI:** 10.3390/brainsci13040536

**Published:** 2023-03-24

**Authors:** Cheng Tan, Jianzhong Ai, Ye Zhu

**Affiliations:** West China Hospital, Sichuan University, Chengdu 610041, China

**Keywords:** mTORC1-dependent protein, Parkinson’s disease, Mendelian randomization, EIF4EBP, EIF4G

## Abstract

Background: The mTOR pathway is crucial in controlling the growth, differentiation, and survival of neurons, and its pharmacological targeting has promising potential as a treatment for Parkinson’s disease. However, the function of mTORC1 downstream proteins, such as RPS6K, EIF4EBP, EIF-4E, EIF-4G, and EIF4A, in PD development remains unclear. Methods: We performed a Mendelian randomization study to evaluate the causal relationship between mTORC1 downstream proteins and Parkinson’s disease. We utilized various MR methods, including inverse-variance-weighted, weighted median, MR–Egger, MR-PRESSO, and MR-RAPS, and conducted sensitivity analyses to identify potential pleiotropy and heterogeneity. Results: The genetic proxy EIF4EBP was found to be inversely related to PD risk (OR = 0.79, 95% CI = 0.67–0.92, *p* = 0.003), with the results from WM, MR-PRESSO, and MR-RAPS being consistent. The plasma protein levels of EIF4G were also observed to show a suggestive protective effect on PD (OR = 0.85, 95% CI = 0.75–0.97, *p* = 0.014). No clear causal effect was found for the genetically predicted RP-S6K, EIF-4E, and EIF-4A on PD risk. Sensitivity analyses showed no significant imbalanced pleiotropy or heterogeneity, indicating that the MR estimates were robust and independent. Conclusion: Our unbiased MR study highlights the protective role of serum EIF4EBP levels in PD, suggesting that the pharmacological activation of EIF4EBP activity could be a promising treatment option for PD.

## 1. Introduction

The prevalence of Parkinson’s disease (PD), an age-related neurodegenerative condition, is increasing at a staggering rate [1]. In addition to decreased dopamine levels, it is typified by a progressive loss of dopaminergic neurons in the substantia nigra pars compacta, which leads to symptoms such as tremors, muscle stiffness, and bradykinesia. The accumulation of α-synuclein (α-syn), the major component of Lewy inclusion, has also emerged as a prominent neuropathological presentation of PD [2,3]. Unfortunately, there is currently no cure for PD. The current treatment strategies focus on managing symptoms and preventing neuronal death, making the identification of new therapeutic targets for PD an urgent priority. Understanding the exact causes and mechanisms of the disease will aid in this effort.

There is growing evidence that mTOR plays a key role in modulating the proliferation, differentiation, and survival of neurons [4]. The mTOR protein kinase is widely present in all types of mammalian cells and plays a crucial role in various cellular processes, including protein synthesis, establishment of the cytoskeleton structure, metabolic regulation, cell growth, and survival [5,6]. mTOR exists in two distinct complexes: mTOR complex 1 (mTORC1) and mTOR complex 2 (mTORC2). mTORC1 is more responsive to rapamycin, the primary inhibitor of mTOR, than mTORC2 [7]. Various stimuli, such as alterations in the levels of insulin, energy, and amino acids, are perceived by mTORC1, which is frequently involved in protein synthesis, cell growth, cell proliferation, and autophagy. mTORC2, on the other hand, plays a role in cytoskeletal rearrangements [8,9]. Major targets of mTORC1 include ribosomal protein S6K kinase 1 (RPS6K) and eukaryotic initiation factor 4E-binding proteins (EIF4EBPs). Both of them are the main regulators of cap-dependent protein synthesis and exert their effects on the regulation of protein translation by modulating transcript initiation and elongation as well as ribosome biosynthesis [5,10]. Meanwhile, the eIF4F complex (eIF4E, eIF4G, and eIF4A) is essential for cap-dependent protein translation, which is a rate-limiting step in protein synthesis. RPS6K is a positive regulator of eIF4F, while EIF4EBP tightly binds to eIF4E and negatively modulates eIF4F complex activity. Once EIF4EBP is phosphorylated by mTORC1, the dissociation of eIF4E–EIF4EBP initiates 5′ cap-dependent mRNA translation [11,12,13]. mTOR is thought to be involved in neurodegenerative disorders through its effects on autophagy and protein synthesis.

In such disorders, an abnormal mTOR signaling cascade can disrupt autophagy flux, leading to the accumulation of protein aggregates and hampering neuron survival [14]. As a critical regulator involved in cell metabolism and survival, mTOR has been considered a promising target against Parkinson’s disease. However, the role of mTOR in PD seems to be controversial, as both neuroprotective and neurotoxic effects have been observed in different PD models. The neuroprotective effects induced by mTOR activation seem to contradict the benefits of inhibiting mTOR in PD [15]. Therefore, we urgently want to know whether there is a causal relationship between mTORC1 downstream proteins and Parkinson’s disease, and whether this causality is protective or pathogenic.

Mendelian randomization (MR) analysis is a novel epidemiological method that uses summary statistics from genome-wide association studies (GWASs) to infer causality between exposures and certain diseases and identify potential risk factors. By using genetic variants as instrumental variables for exposures, MR can overcome the confounding factors that are often present in observational studies [16,17]. In this study, we used two-sample MR based on GWAS summary statistics of mTORC1-associated proteins in the European population to investigate the causal association of mTORC1-associated proteins (RPS6K, EIF4EBP, EIF-4E, EIF-4G, and EIF4A) with PD.

## 2. Materials and Methods

### 2.1. Data Sources for Exposure

The genetic variation of mTORC1-related proteins was extracted from the publicly available Human Plasma Proteome Atlas. The atlas includes data on 3622 plasma proteins obtained from 3301 healthy participants undergoing genetic control from the INTERVAL study [18]. These data link genetic factors to phenotypes based on protein information, providing a basis for potential targets for certain diseases. The INTERVAL study enrolled approximately 50,000 participants who were 18 years and older and excluded those with major diseases. The recruitment took place at 25 centers of England’s National Health Service Blood and Transplant (NHSBT) from mid-2012 to mid-2014. Blood samples were collected in EDTA tubes after standard venipuncture, and the isolated plasma was stored at −80 °C for research use [19]. The relative concentrations of 3622 plasma proteins were measured using the SOMAscan assay method, which can simultaneously measure extracellular and intracellular proteins as well as expand the lower limit of detection for protein abundance [20]. After a natural log transformation and adjustment for age, sex, and duration between the blood draw and linear regression processing, the protein concentrations could be determined [18].

### 2.2. Data Sources for the Outcome

The summary statistics for genetic studies on PD were gathered by the International Parkinson’s Disease Genomics Consortium. The data include 33,674 individuals diagnosed with PD and 449,056 controls from European ancestry samples. The analysis was a fixed-effects meta-analysis across 17 datasets that all underwent a similar process of quality control for inclusion [21].

### 2.3. Instrumental Variable

**Figure 1:**
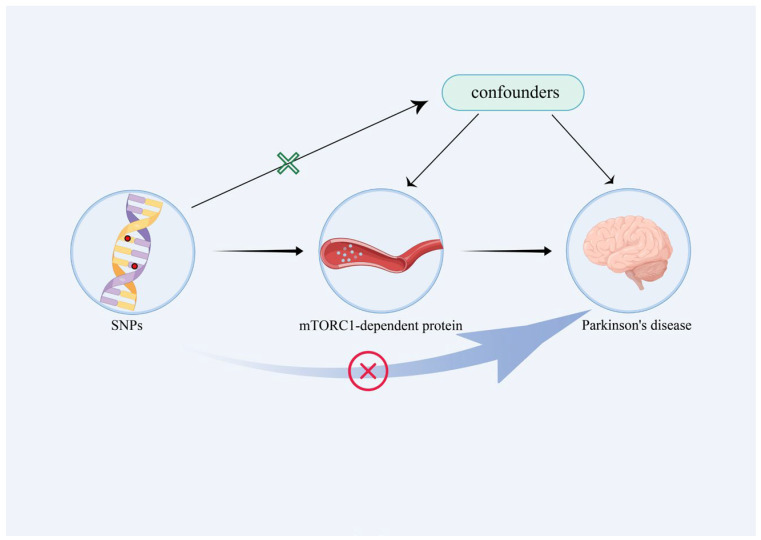
Illustrative depiction of the MR study design. SNP, single-nucleotide polymorphism.

We established inclusion criteria for selecting instrumental variables in order to obtain a precise assessment. These criteria include a significance threshold of *p* < 5 × 10^−6^ and a linkage disequilibrium (LD) r2 < 0.05. Additionally, single-nucleotide polymorphisms (SNPs) with palindromic sequences were removed from the analysis. The acquired instrumental variables were evaluated to ensure that they met the fundamental assumptions of a Mendelian randomization analysis (Figure 1): (1) they had to be significantly associated with the exposure of interest; (2) they must not have any relationship with potential confounders, and (3) the association between the instrumental variables and PD must be mediated by the exposure. We also utilized PhenoScanner to verify if any potential confounders were linked to the chosen instrumental variables by the SNPs. Ultimately, we identified reliable instrumental variables as proxies for the following proteins: RPS6K (16 SNPs), EIF4EBP (6 SNPs), EIF4E (13 SNPs), EIF4A (10 SNPs), and EIF4G (7 SNPs).

### 2.4. Mendelian Randomization Analyses

We applied several MR methods including inverse-variance-weighted (IVW), weighted median (WM), and MR–Egger to determine the causal effects between mTORC1-related proteins and PD. The IVW results were used as primary information, and a meta-analysis was performed to aggregate the Wald estimates of single SNPs in order to obtain overall estimates of mTORC1-related proteins on PD, respectively. These unbiased results were based on the assumption that there was no horizontal pleiotropy [22]. MR–Egger [23] and weighted median [24] analyses were used as supplemental evidence for the IVW results as they provide more robust estimates under a broad range of scenarios, despite lower efficiency. The MR-RAPS method can weigh the instrumental variables based on their strength, which improves the efficiency of MR estimates in the presence of weak instrumental variables while also balancing pleiotropy [25]. Additionally, sensitivity analyses were conducted to detect potential pleiotropy and heterogeneity. Heterogeneity was reported via Cochran’s Q test (*p* < 0.05). The intercept of MR–Egger regression provided evidence to evaluate horizontal pleiotropy (*p* < 0.05) [26]. MR pleiotropy residual sum and outlier (MR-PRESSO) identified biased variants if they existed and removed them progressively to reduce any sign of horizontal pleiotropy [27]. Finally, the leave-one-out method was used to test whether any individual SNP confounded the effect estimates, for more robust conclusions. All the statistical analyses were carried out using the TwoSampleMR (version 0.5.6) and MR-PRESSO (version 1.0) packages in R (version 4.2.1). The *p*-value threshold for Bonferroni correction was set at 0.01 (0.05/5) to adjust for multiple testing. Results with a significance level of less than 0.01 were considered strong evidence of a causal relationship, while those with a significance level between 0.01 and 0.05 were considered indicative evidence of a relationship.

## 3. Results

**Figure 2:**
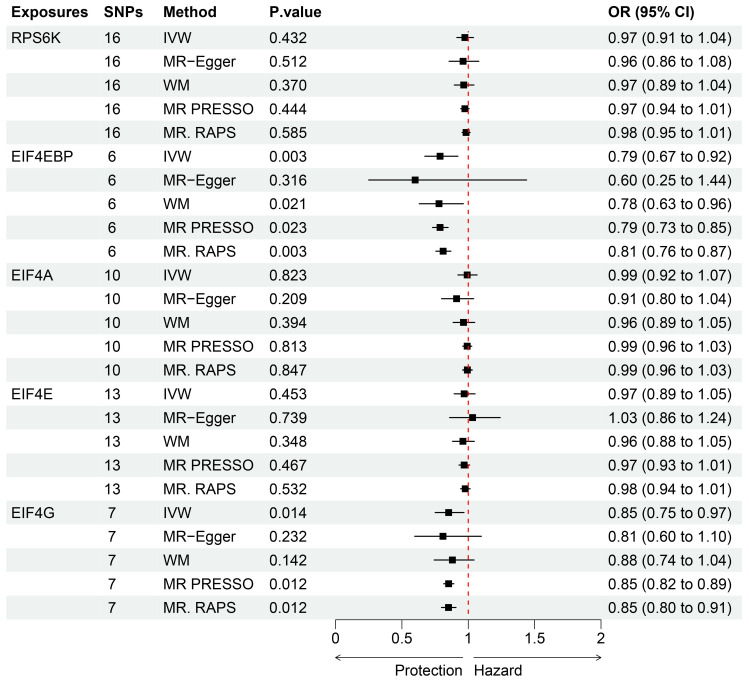
Associations between genetic proxies of mTORC1-dependent proteins and Parkinson’s disease analyzed using various MR models. SNP, single-nucleotide polymorphism; OR, odds ratio; CI, confidence interval; IVW, inverse-variance-weighted; WM, weighted median; MR-RAPS, robust adjusted profile score; MR-PRESSO, MR pleiotropy residual sum and outlier; RP-S6K, ribosomal protein S6K kinase; EIF4EBP, eukaryotic initiation factor 4E-binding protein; EIF-4G, translation initiation factor 4G; EIF-4E, translation initiation factor 4E; EIF-4A, translation initiation factor 4A.

**Figure 3:**
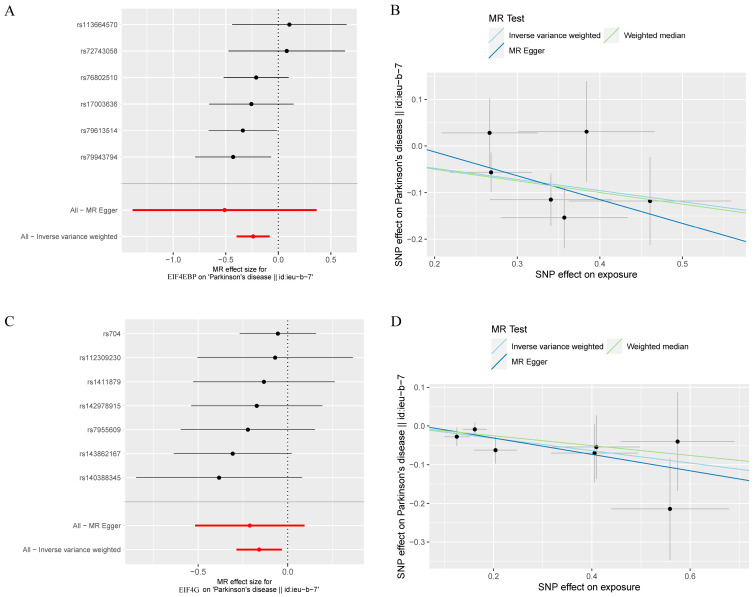
MR analyses of genetic proxied EIF4EBP and EIF4G levels on the risk of PD. The forest plot (**A**) and the scatterplot (**B**) of the MR analysis of EIF4EBP and PD. The forest plot (**C**) and the scatterplot (**D**) of the MR analysis of EIF4G and PD.

**Figure 4:**
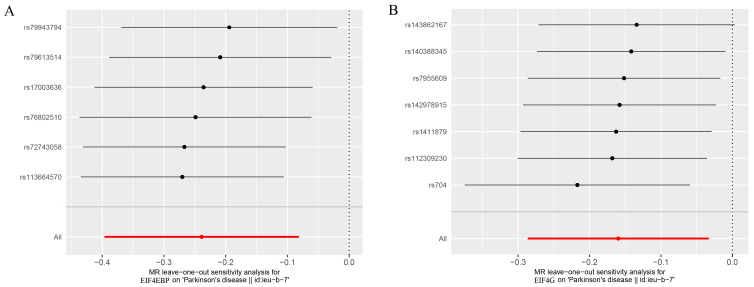
Leave-one-out analysis for the estimates of EIF4EBP (**A**) and EIF4G (**B**) on PD.

As seen in Figure 2, the levels of the genetic proxy EIF4EBP were found to have an inverse association with the risk of PD (OR = 0.79, 95% CI = 0.67–0.92, *p* = 0.003). The results obtained from WM, MR-PRESSO, and MR-RAPS were consistent, which added confidence to the conclusion that this association is likely causal. Additionally, indicative evidence was found for a potential causal effect of EIF4G levels on the risk of PD, whereby higher levels reduced the risk (OR = 0.85, 95% CI = 0.75–0.97, *p* = 0.014). Similar causal estimates were also obtained using MR-PRESSO (OR = 0.85, 95% CI = 0.82–0.89, *p* = 0.012) and MR-RAPS (OR = 0.85, 95% CI = 0.80–0.91, *p* = 0.012). The detailed instrumental variables in the analysis were listed (Table S1). The impact of individual variables of EIF4EBP and EIF4G on PD was further demonstrated through the scatterplot and forest plot presented in Figure 3. However, the analysis of the effects of RPS6K, EIF4A, and EIF4E levels on the risk of PD did not reach statistical significance. The Cochran’s Q test *p*-values for all of these were more than 0.05, indicating that no obvious heterogeneity was observed. Furthermore, the MR–Egger regression intercept showed no evidence of unbalanced pleiotropy (all *p* > 0.05) (Table 1). The global test of MR-PRESSO also did not identify any pleiotropic bias. In addition, the absence of any significant SNPs in the leave-one-out sensitivity analysis (Figure 4) suggests that no individual SNP could disrupt the causal relationship.

## 4. Discussion

As far as we know, this study is the initial investigation into the causal connection between the levels of mTORC1-dependent proteins (including RPS6K, EIF4EBP, EIF-4E, EIF-4A, and EIF-4G) and PD using MR methods and eliminating potential confounding factors through genetic variations. Utilizing data from the INTERVAL study on human plasma protein arrays and GWAS summary statistics from the International Parkinson’s Disease Genomics Consortium (IPDGC), we conducted a two-sample MR analysis. The MR study indicated a negative correlation between the levels of the EIF4EBP protein in the plasma (OR = 0.79, 95% CI = 0.67–0.92, *p* = 0.003) and PD risk. We also discovered suggestive evidence of a protective effect of the plasma protein levels of EIF4G on PD (OR = 0.85, 95% CI = 0.75–0.97, *p* = 0.014), despite the *p*-value not being significant after Bonferroni correction. However, no clear evidence was found to support a causal effect of the gene-predicted RP-S6K, EIF-4E, and EIF-4A on PD risk. Sensitivity analyses also did not reveal any significant imbalanced pleiotropy or heterogeneity, indicating that the MR estimates were independent and robust.

The mechanism by which mTOR regulates the survival of dopamine-producing neurons is still largely unknown. The inhibition of mTORC1 with rapamycin has been shown to decrease the levels of PARK7/DJ1, a protein linked to neurodegeneration that has beneficial molecular chaperone and antioxidant properties and whose translation depends on mTORC1. Loss-of-function mutations in PARK7 are associated with early-onset, recessive forms of Parkinson’s disease [28]. The mTOR signaling pathway is essential for regulating protein synthesis and cell survival; thus, it is important to keep it active for its protective role in neurons. However, the fine-tuning of mTOR activation may be necessary during Parkinson’s disease. mTORC1 controls both protein synthesis through translation and protein degradation through autophagy. It negatively regulates autophagy by phosphorylating Atg13 and ULK1/2, inhibiting the formation of autophagosomes [29,30]. Alpha-synuclein accumulation is a hallmark of Parkinson’s disease, and it is believed to play a key role in the death of dopamine-producing neurons. By inhibiting mTOR, rapamycin promotes autophagy, which is a process by which cells degrade and recycle waste proteins and damaged cellular components. By increasing autophagy, rapamycin may help to prevent the accumulation of toxic alphasynuclein and protect dopamine-producing neurons from death. This highlights the potential therapeutic value of mTOR inhibition for treating Parkinson’s disease [31]. In summary, excessive or inadequate mTOR activity can be lethal to neurons.

EIF4EBP is a crucial component of the mTORC1 signaling pathway, and its activity is tightly regulated by the balance of its phosphorylation levels. When EIF4EBP is underphosphorylated, it binds strongly to eIF4E, thus inhibiting cap-dependent translation initiation. However, when excessive phosphorylation occurs, EIF4EBP is released from eIF4E, resulting in increased cap-dependent translation [32,33]. The postmortem analysis of the brains of PD patients suggests oxidative stress as a potential mechanism of neurodegeneration. This is indicated by a lack of the antioxidant glutathione (GSH) and the inhibition of mitochondrial complex I activity in the substantia nigra, along with increased superoxide dismutase (SOD) activity [34]. It has been proven that EIF4EBP is crucial for survival during stressful conditions, including starvation and oxidative stress. Activated EIF4EBP induces a rapid halt in cap-dependent translation, promoting the upregulation of stress response factors, such as antioxidants and molecular chaperones [35,36,37]. CHCHD2 is a mitochondrial protein-encoding gene that controls oxidative phosphorylation by regulating Cyt c and crista integrity. Dominant mutations in this gene are the cause of late-onset PD [38]. In a limited case study, several exonic variants that may affect CHCHD2 protein levels or subcellular localization showed an association with Parkinson’s disease [39]. In Drosophila, the loss of CHCHD2 can lead to abnormalities in the mitochondrial matrix structure and impaired mitochondrial respiration, which exacerbates the sensitivity to oxidative stress, loss of dopaminergic neurons, and motor dysfunction associated with Parkinson’s disease. In addition to these PD pathological phenotypes, a compensatory upregulation of 4E-BP was also observed. These PD-related phenotypes were alleviated by the overexpression of the translation inhibitor 4E-BP and the introduction of human CHCHD2. By introducing 4E-BP into dCHCHD2-/-Drosophila, the general expression of 4E-BP decreased the ATP decline in drosophila. The same outcome was achieved by administering rapamycin, which activates 4E-BP by inhibiting the kinase target of rapamycin [40]. Mitochondrial dysfunction is a prominent feature of PD, and the beneficial role of 4E-BP in mitochondria reverses PD pathological phenotypes. Additionally, mutations in PINK1 and parkin lead to autosomal recessive hereditary Parkinson’s disease. Drosophila with PINK1 and parkin mutations display dopaminergic neurodegeneration, motor defects, and mitochondrial dysfunction. These mutations are associated with significant reductions in the phosphorylated levels of EIF4EBP, with corresponding increases in the proportion of active nonphosphorylated EIF4EBP. The over-expression of EIF4EBP can suppress all pathological phenotypes, and the loss of EIF4EBP function significantly reduces the survival ability of parkin and PINK1 mutants. In vivo, EIF4EBP can be activated by the TOR inhibitor rapamycin [41]. Moreover, LRRK2 homologous mutations in Drosophila can produce PD phenotypes similar to those caused by parkin/PINK1 mutations, the most common genetic cause of PD [42]. LRRK2 contains both an active GTPase and kinase domain, which can promote cap-dependent translation and exhibit strong genetic interactions with the core members and regulators of the cap-binding protein complex. Generally, disease-associated mutations in LRRK2 often increase its kinase activity, thereby enhancing its toxicity. To date, a large number of LRRK2 mutations have been identified, with the G2019S variant being the most common. This mutation leads to increased protein synthesis in neurons, exhibiting the age-related loss of dopaminergic neurons and motor dysfunction, which can be improved by blocking the synthesis with protein synthesis inhibitors [43,44]. LRRK2 acts as a protein translation regulator by phosphorylating the 4E-BP protein at the T37/T46 site both in vitro and in vivo. However, the control of translation initiation is closely related to stress and lifespan, and these phosphorylation events appear to have important effects on stress sensitivity and dopaminergic neuron survival in LRRK2 mutant Drosophila. On the one hand, the overexpression of eIF4E and dLRRK leads to age-related phenotypes in DA neurons, whose gene expression pattern is consistent with gene expression under oxidative stress and aging conditions. This strongly suggests that a chronic decline in 4E-BP activity promotes oxidative stress and subsequent aging in DA neurons. On the other hand, it has been observed that the overexpression of 4E-BP or a reduction in dLRRK levels can provide protection in Drosophila models of PINK1 and parkin pathology [41,44,45]. This suggests that regulating 4E-BP expression to suppress the accumulation of abnormal protein is beneficial. In our study, we revealed an inverse association between the levels of the genetic proxy EIF4EBP and PD risk. This is consistent with the observed effects in experimental studies.

The expression of specific target genes and protein levels in circulation can be modulated, making them potential targets for preventing PD. Given the mounting evidence implicating the mTOR signaling pathway in the pathogenesis of PD, it is unclear whether there is a causal relationship between mTORC1-related proteins and PD. Using epidemiological research methods, we determined in this study that an increase in EIF4EBP protein levels can help prevent PD, as previously observed in experimental studies. The disclosure of this unbiased causal relationship will guide a direction for future PD prevention and provide a potential intervention target, while also requiring further basic research to uncover potential pathophysiological mechanisms. In conclusion, targeting the mTOR pathway pharmacologically has promising potential as a treatment for PD. Further work is needed to fully understand the nuanced regulation of mTOR and its complex cellular pathways. It is crucial to continue the development of mTOR signaling modulators that meet therapeutic goals, while mitigating the adverse effects of current mTOR regulators, to achieve the desired clinical outcomes without negative consequences. The pharmacologic activation of EIF4EBP activity may represent a viable treatment option for PD.

One advantage of this study is the use of MR design. As alleles are randomly assigned and fixed at conception, it minimizes the potential for observed associations to be biased by reverse causation and confounding. Secondly, we employed various MR methods including IVW, MR–Egger, WM, and MR-RAPS to robustly estimate causal relationships. MR–Egger regression and MR-PRESSO were used to detect pleiotropy to ensure the robustness of the conclusions. Thirdly, we provided robust genetic instruments by using multiple SNPs as instrumental variables for mTORC1-related proteins. Fourthly, our analysis utilized a large sample size with no overlap between the exposure and outcome, thereby resulting in high statistical power for most of the analyses. This study has several limitations. Firstly, all the GWAS data were from individuals of European ancestry, and it remains to be determined whether the results observed in this study are generalizable to other populations. Therefore, future studies utilizing MR to investigate the causal relationship between mTORC1-dependent proteins (including RPS6K, EIF4EBP, EIF-4E, EIF-4A, and EIF-4G) and PD should consider including samples from different ethnic groups to increase the generalizability of the findings. Secondly, as the study used summary statistics data and not individual-level data, it was not possible to conduct a subgroup analysis. Thirdly, the genetic variants used in MR studies reflect lifetime exposure and specific causal associations, whereas randomized controlled trials only report the effects of short-term exposure. Therefore, it is difficult to determine the effects of short-term exposure to high levels of EIF4EBP proteins on PD risk and to understand the effectiveness of any interventions.

## 5. Conclusions

This unbiased MR study highlights the protective role of serum EIF4EBP levels in PD. Further research is required regarding the study of EIF4EBP’s role. Nevertheless, this study’s findings lay the foundation for a more effective pharmacological target in preventing and treating PD.

## Figures and Tables

**Table 1:** Sensitivity analysis for the association between mTORC1-dependent protein and PD.

Exposure	Cochran’s Q Test		MR–Egger		MR-PRESSO	
	Q Value	*p* Value	Egger Intercept	*p* Value	Global Test	*p* Value
RPS6K	15.53	0.41	0.0049	0.78	16.60	0.51
EIF4EBP	4.27	0.51	0.0895	0.57	5.63	0.60
EIF4A	7.64	0.57	0.0226	0.17	12.74	0.50
EIF4E	15.31	0.23	−0.0228	0.47	16.61	0.32
EIF4G	2.88	0.82	0.0111	0.73	4.68	0.79

RP-S6K, ribosomal protein S6K kinase; EIF4EBP, eukaryotic initiation factor 4E-binding protein; EIF-4G, translation initiation factor 4G; EIF-4E, translation initiation factor 4E; EIF-4A, translation initiation factor 4A.

## Data Availability

All data used for this study are publicly available.

## Supplementary Materials

The following supporting information can be downloaded at: https://www.mdpi.com/article/10.3390/brainsci13040536/s1. Table S1: The instrumental variables associated with exposure.
