# Supplementary material for: mTORC1-Dependent Protein and Parkinson’s Disease: A Mendelian Randomization Study
Source: Brain Sci. 2023 Mar 24;13(4):536. doi: 10.3390/brainsci13040536 (PMC10137243; doi:10.3390/brainsci13040536)
Supplement: Supplementary file 1 [file brainsci-13-00536-s001.zip › brainsci-2260047-supplementary.pdf]

brain sciences

MDPI

Article

mTORC1−dependent protein and Parkinson's disease: A Mendelian randomization study

Table S1. The instrumental variables associated with exposure.

Exposure	SNP	Effect allele	Other allele	Beta.exposure	Beta.outcome	Se.exposure	Se.outcome
**RPS6K**	rs1355191	C	T	0.125	−0.0263	0.0263	0.0229
	rs1381968	T	C	0.3462	0.0871	0.0746	0.0711
	rs138831183	G	A	−0.5177	0.151	0.1074	0.1384
	rs148800371	T	G	0.1637	0.0711	0.0327	0.0356
	rs148897689	G	A	−0.4496	0.0266	0.0943	0.0968
	rs35747952	T	C	0.3736	−0.0042	0.0809	0.0744
	rs3859503	A	C	−0.1892	0.044	0.0333	0.0322
	rs58565824	C	T	−0.4607	0.1101	0.0937	0.0875
	rs62143197	G	A	−0.5347	0.0182	0.029	0.022
	rs62398809	A	G	0.1129	4.00E−04	0.0247	0.0224
	rs7017005	A	G	0.1213	−0.0099	0.0256	0.0227
	rs72881486	G	A	−0.3633	0.0371	0.0745	0.0785
	rs75688971	T	C	−0.4179	0.0505	0.0836	0.0899
	rs77394885	A	C	−0.5291	−0.0063	0.1122	0.0986
	rs79777011	C	A	−0.1634	−0.0358	0.0346	0.0312
	rs9833044	C	T	0.319	0.0833	0.0635	0.0603
**EIF4EBP**	rs113664570	C	T	−0.2666	−0.0281	0.0584	0.0741
	rs17003636	T	C	−0.4607	0.1179	0.0984	0.0944
	rs72743058	G	A	0.3839	0.0308	0.0827	0.1086
	rs76802510	C	T	0.2684	−0.0565	0.0496	0.0424
	rs79613514	T	C	−0.3407	0.1149	0.0738	0.0566
	rs79943794	C	T	0.357	−0.1534	0.0766	0.0658
**EIF4A**	rs11084300	C	T	−0.1431	0.0085	0.0272	0.0302
	rs1447676	C	T	0.1182	0.0371	0.0257	0.0229
	rs145852535	A	G	−0.521	−0.032	0.1118	0.1932
	rs1931094	G	A	−0.1163	0.0021	0.0253	0.0201
	rs2462049	G	T	−0.1159	−0.0068	0.0249	0.0225
	rs34131899	G	A	0.2829	0.0053	0.0591	0.0514
	rs34436714	C	A	−0.4687	0.0203	0.0291	0.0214
	rs3859507	G	T	−0.1777	0.0394	0.0327	0.0313
	rs6792693	A	G	−0.134	−0.0255	0.0286	0.0203
	rs74512707	G	A	−0.1785	−0.0304	0.0385	0.0344
**EIF4E**	rs11084300	C	T	−0.1585	0.0085	0.0272	0.0302
	rs116934738	T	C	−0.3758	−0.0392	0.077	0.0784

	rs138236097	G	A	0.2673	−0.0639	0.0583	0.0556
	rs142569846	G	T	−0.2303	−0.0429	0.0468	0.0429
	rs149036167	T	C	0.5168	−0.1404	0.108	0.1165
	rs192028145	A	G	0.5295	−0.1454	0.1025	0.1056
	rs192206210	T	G	−0.5846	−0.1307	0.1211	0.1213
	rs2209485	C	T	−0.1637	0.0574	0.034	0.0321
	rs62130614	G	T	−0.4606	−0.1956	0.0999	0.0912
	rs62143198	G	A	−0.4669	0.019	0.0296	0.022
	rs741454	T	C	−0.1426	0.0132	0.031	0.0281
	rs74842834	G	T	−0.4443	−0.0238	0.0947	0.0949
	rs76641346	G	T	−0.3052	−0.0126	0.063	0.0625
**EIF4G**	rs112309230	T	C	0.5745	−0.0404	0.1158	0.1273
	rs140388345	G	A	−0.559	0.2141	0.1201	0.132
	rs1411879	G	A	−0.4092	0.0544	0.089	0.0825
	rs142978915	T	C	0.4055	−0.0701	0.0885	0.0757
	rs143862167	C	T	0.204	−0.0626	0.044	0.0342
	rs704	G	A	−0.1618	0.0089	0.0245	0.0176
	rs7955609	G	A	−0.1251	0.0278	0.0263	0.0239
